# Dietary Fats and Depressive Symptoms in Italian Adults

**DOI:** 10.3390/nu15030675

**Published:** 2023-01-28

**Authors:** Walter Currenti, Justyna Godos, Amer M. Alanazi, Giuseppe Lanza, Raffaele Ferri, Filippo Caraci, Fabio Galvano, Sabrina Castellano, Giuseppe Grosso

**Affiliations:** 1 Department of Biomedical and Biotechnological Sciences, University of Catania, 95123 Catania, Italy; 2 Department of Pharmaceutical Chemistry, College of Pharmacy, King Saud University, P.O. Box 2457, Riyadh 11451, Saudi Arabia; 3 Clinical Neurophysiology Research Unit, Oasi Research Institute-IRCCS, 94018 Troina, Italy; 4 Department of Surgery and Medical-Surgical Specialties, University of Catania, 95123 Catania, Italy; 5 Sleep Research Centre, Department of Neurology IC, Oasi Research Institute-IRCCS, 94018 Troina, Italy; 6 Neuropharmacology and Translational Neurosciences Research Unit, Oasi Research Institute-IRCCS, 94018 Troina, Italy; 7 Department of Drug and Health Sciences, University of Catania, 95125 Catania, Italy; 8 Department of Educational Sciences, University of Catania, 95124 Catania, Italy; 9 Center for Human Nutrition and Mediterranean Foods (NUTREA), University of Catania, 95123 Catania, Italy

**Keywords:** dietary fats, fat, monounsaturated fats, polyunsaturated fats, saturated fats, short-chain fatty acids, depression, mood, arachidonic acid

## Abstract

Background: Depression represents one of the major causes of disability worldwide, with an important socioeconomic cost. Although many risk factors have been considered in its pathogenesis, nutrition seems to play a determinant role in its prevention. With regard to individual macronutrients, dietary fats and especially n-3 polyunsaturated fatty acids (n-3 PUFA) are the most studied. However, previous data about other dietary fatty acids, such as n-6 PUFA, are conflicting, and little is known about saturated fatty acids (SFA), especially when considering carbon chain length. Thus, we investigated whether single types and subtypes of dietary fats are related to depressive symptoms in Italian individuals living in the Mediterranean area. Methods: Dietary and socio-demographic data of 1572 individuals were analyzed. Food frequency questionnaires (FFQs) were used to determine the consumption of total dietary fat and each specific class of dietary fat, such as SFA, monounsaturated fatty acid (MUFA), and PUFA. The intake of fatty acids was also assessed according to the carbon-chain length of each single class. The Center for Epidemiologic Studies Depression Scale (CES-D) was used as a screening tool for depressive symptoms. Results: After adjustment for potential confounding factors, a significant inverse association between low/moderate levels of PUFA intake and depressive symptoms (Q2 vs. Q1, odds ratio (OR) = 0.60, 95% CI: 0.44, 0.84) was found. On the other hand, moderate saturated fat consumption was associated with depressive symptoms (Q3 vs. Q1, OR = 1.44, 95% CI: 1.02, 2.04). However, when considering carbon chain length, individuals with a lower to moderate intake of short-chain saturated fatty acids (SCSFA) and medium-chain saturated fatty acids (MCSFA) were less likely to have depressive symptoms (Q3 vs. Q1, OR = 0.48, 95% CI: 0.31, 0.75), while moderate intake of arachidic acid (C20:0) was directly associated with depressive symptoms (Q3 vs. Q1, OR = 1.87, 95% CI: 1.26, 2.77). Among single MUFAs, higher myristoleic acid (C14:1) intake was directly associated with depressive symptoms (Q4 vs. Q1, OR = 1.71, 95% CI: 1.12, 2.61), while moderate intake of erucic acid (C22:1) was associated with lower odds of having depressive symptoms (Q3 vs. Q1, OR = 0.54, 95% CI: 0.33, 0.86). When considering individual PUFAs, individuals with moderate and higher intakes of arachidonic acid (C20:4) were less likely to have depressive symptoms (OR = 0.64, 95% CI: 0.45, 0.91; OR = 0.59, 95% CI: 0.38, 0.91, respectively). Similarly, higher eicosapentaenoic acid (C20:5) intake was inversely associated with depressive symptoms (Q4 vs. Q1, OR = 0.35, 95% CI: 0.12, 0.98), while a significant association for docosahexaenoic acid (C22:6) was retrieved only for low intakes (Q2 vs. Q1, OR = 0.33, 95% CI: 0.12, 0.88). Conclusions: Dietary fat intake may be associated with depressive symptoms, underlying the importance of distinguishing between different fat types. This study confirms the pivotal role of PUFAs and reopens the debate on the role of saturated fatty acids, suggesting plausible effects of moderate intakes of short-chain fatty acids.

## 1. Introduction

Sadness, low self-worth, alterations in sleep and appetite, a loss of interest or pleasure in daily activities, and even recurring thoughts of suicide are common symptoms of depression. A recent World Health Organization (WHO) report showed that depression affects over 300 million people in the world [1,2,3] and represents one of the main causes of disability [4], with an impactful socio-economic cost weighing on our health systems [5]. This data is more alarming considering that during the COVID-19 pandemic, there was an increase of 50 million (+30%) cases globally [1]. The pathophysiology of depression is multifactorial and not yet fully elucidated. Although there is a strong genetic component, the external environment plays a determinant role in the onset of depression; abuse, a lower socioeconomic level, poor education, marital status, chronic stress, racial discrimination, and disabilities are the most relevant social contributors [6]. Among environmental factors, nutrition seems to play a key role in the pathophysiology of depression and potentially also in its treatment. Recent studies showed that the consumption of high-calorie density foods (such as refined grains, sugar, cooking oils, corn syrup, and ultra-processed foods) increases the odds of becoming depressed [7,8,9,10], while on the contrary, higher adherence to traditional plant-based Mediterranean patterns (rich in fruits, vegetables, nuts, fish, and olive oils) prevents depression [11]. This increased interest in the role of nutrition on mental health has given rise to “nutrition psychiatry”, which studies how dietary patterns but also individual foods, macro/micronutrients and bioactive compounds are related to mental disorders [12,13]. For example, lower levels of calcium, magnesium, iron, zinc, vitamin D, and many of the B vitamins are typically found in subjects with depression [14], and lower levels of potassium, phosphorus, and copper are associated with the severity of symptoms [15]. Beverages, like tea and coffee, as well as their bioactive compounds, have been shown to exert neuroprotective effects by reducing the risk of brain disorders [16,17,18]. Regarding macronutrients, it seems that higher intakes of carbohydrates and lower consumptions of protein are associated with depression [19], although in the literature, dietary fats are the most studied macronutrients regarding mood disorders. The role of polyunsaturated fats (PUFA) in fact was widely studied both in the prevention and in the treatment of depression. It seems that n-3 PUFA may prevent depression by exerting anti-inflammatory properties, modulating neuroendocrine pathways, and activating crucial neurotransmitters [20]. Although previous data showed that dietary n-3 PUFA intake is associated with a lower risk of depression [21,22,23], a recent meta-analysis showed that n-3 PUFA supplementation probably has little or no effect on preventing depression and its typical symptoms [24]. Nonetheless, less is known regarding the link between the intake of other fatty acids, such as monounsaturated fatty acids (MUFA) or saturated fatty acids (SFA), and the risk of depression. In a prospective study that involved 120,000 Spanish adults, high trans unsaturated fatty acid (TFA) consumption was associated with depression risk, while only weak inverse associations were found for MUFA, especially from olive oil [9]. Regarding SFA, studies on animals showed that a high intake of these fatty acids stimulates pro-inflammatory cytokine production and apoptosis in astrocytes, resulting in neuroinflammation, that impairs the dopamine system and increases the risk of depression [25]. A recent prospective study showed that high SFA intake was linked with the presence of depressive symptoms in midlife women [26], while another cross-sectional study failed to find a statistically significant association after adjustment for total caloric intake [27]. A possible cause for these disputed results may originate from the type of SFA consumed in the diet. In fact, SFA differ from each other according to the length of the carbon chain: short-chain saturated fatty acids (SCSFAs, from 2 to 6 carbons), medium-chain saturated fatty acids (MCSFAs, from 8 to 12 carbons), and long-chain saturated fatty acids (LCSFAs, from 14 to 20 or more carbons) [28]. Interestingly, recent data showed possible beneficial effects of SCSFA on health [29], thanks to their anti-inflammatory properties [30] and their pivotal role in microbiota-gut-brain crosstalk [31]. Thus, the aim of this study was to investigate if specific types and subtypes of dietary fatty acids are linked with depressive symptoms in a Mediterranean sample of Italian adults.

## 2. Materials and Methods

### 2.1. Study Population

The Mediterranean Healthy Eating, Aging, and Lifestyle study, is an observational study aiming to investigate the association between typical lifestyle and dietary habits of the Mediterranean area and non-communicable diseases. Between 2014 and 2015, a cohort of 2044 randomly selected individuals (≥18 years old) from Catania, in the south of Italy, was recruited for the study, the protocol for which is published elsewhere [32]. All the individuals enrolled in the study gave written informed consent and were aware of the research’s purpose. All project processes were carried out in compliance with the Declaration of Helsinki (1989). The study protocol was reviewed and approved by the ethical committee.

### 2.2. Data Collection

Presence-assisted interviews, supported by tablet computers, were used as a data collection method. Moreover, a paper copy of the questionnaire was distributed to all subjects, allowing them to visualize each response option. The answers were immediately registered by the interviewer. The demographic data, including sex, age at recruitment, and educational status, were collected. Educational status was classified as: (i) low (primary/secondary), (ii) medium (high school), and (iii) high (university). The International Physical Activity Questionnaire (IPAQ), consisting of five domains with the aim of investigating the time spent being physically active in the previous week, was used to assess and report motor activity [33]. According to IPAQ, physical activity levels were catalogued into the following categories: (i) low, (ii) moderate, and (iii) high. Smoking status was categorized into (i) non-smoker, (ii) ex-smoker, and (iii) current smoker. The obtaining of anthropometric measurements complied with standard protocols. Subjects were divided by body mass index (BMI) cut-offs as under/normal weight (BMI < 25 kg/m^2^), overweight (from BMI 25 to 29.9 kg/m^2^) and obese (BMI ≥ 30 kg/m^2^) [34].

### 2.3. Depression Assessment

The Center for the Epidemiological Studies of Depression Short Form (CES-D) was the method used to screen the general population for depression symptoms [35]. The occurrence of all symptoms or mood in the last week was rated by each item on the scale, assigning a score ranging from 0 (rare or absent symptom (less than 1 day) to 3 (quite frequent symptom (5–7 days)). The total score, obtained from the sum of all items (after inversion of the positive mood items), can vary from 0 to 30, and higher scores indicate greater severity of the symptoms; a score ≥ 16 suggests depressive states. After excluding subjects with missing data, a total sample of 1572 was finally analyzed.

### 2.4. Dietary Assessment

The dietary assessment was carried out through two food frequency questionnaires (FFQs; a long and a short version) previously validated in the Sicilian population [36,37]. The food composition tables of the Research Center for Foods and Nutrition were used as a comparison tool for the calculation of the energy and macro-micronutrient intake. Data from FFQs were converted to 24 h intake, allowing the calculation in g or ml of the mean daily intake of each food. Then, the total content of specific fatty acids in each food was obtained using Italian food composition tables. A calculation of their daily consumption was achieved by multiplying the content of total and single fatty acid molecules by the daily intake of each food. Adherence to the Mediterranean diet was used as a proxy for diet quality and assessed using a literature-based scoring system. Briefly, two points were given to the highest category of consumption of food groups typical of the Mediterranean pattern (such as vegetables, fruits, legumes, cereals, and fish), one point for the middle category, and 0 points for the lowest category of consumption. Conversely, two points were given for the lowest category of consumption of foods not characteristic of the Mediterranean diet (such as meat and dairy products), one point for the middle category, and 0 points for the highest category of consumption. Better adherence was guaranteed by moderate alcohol intake and regular use of olive oil. The final adherence score includes nine food categories with a score ranging from 0 points (lowest level of adherence) to 18 points (highest level of adherence), and individuals are grouped in tertiles and categorized as low, medium, or high adherents to the Mediterranean diet [38]. FFQs with unreliable intakes (<1000 or >6000 kcal/d) or lacking information were excluded, leaving 1572 individuals.

### 2.5. Statistical Analysis

Continuous variables are reported as means (standard deviations, SDs), while categorical variables are reported as frequencies of occurrence (percentages). Individuals were divided by quartiles of total dietary fat intake, and differences in background characteristics were confronted by the chi-squared test for categorical variables and the ANOVA and Kruskall–Wallis tests for continuous variables that were normally and not normally distributed, respectively. Energy-adjusted and multivariate logistic regression models were performed to assess the relationship between fat consumption and depressive symptoms. The multivariate model was adjusted for background characteristics (age, sex, BMI, physical activity, educational status, smoking status), and adherence to the Mediterranean dietary pattern as a proxy of diet quality. All reported *p*-values were based on two-sided tests and compared to a significance level of 5%. SPSS 17 (SPSS Inc., Chicago, IL, USA) software was used for all the statistical calculations.

## 3. Results

Data from 1572 individuals were analyzed. Table 1 shows the background characteristics of the cohort, grouped by quartiles of total dietary fat intake. Individuals in the highest quartile of dietary fat intake were significantly younger, had higher adherence to the Mediterranean diet, and had lower educational status. Similarly, significant differences were observed in the distribution of physical activity levels, but with no linear trend. No significant differences across quartiles of dietary fat intake were found when considering BMI and smoking status.

Table 2 shows the relationship between total and main classes of dietary fat intake and depressive symptoms. Multivariate-adjusted analysis showed a significant inverse association between low/moderate levels of PUFA intake and depressive symptoms (Q2 vs. Q1, odds ratio (OR) = 0.60, 95% CI: 0.44, 0.84). On the other hand, moderate saturated fat intake was associated with depressive symptoms (Q3 vs. Q1, OR = 1.44, 95% CI: 1.02, 2.04). No associations were found between total fat and MUFA intake and depressive symptoms.

The association between specific sub-classes of fat and depressive symptoms is shown in Table 3. Interestingly, subjects with a lower to moderate intake of SCSFA–MCSFA were less likely to have depressive symptoms (Q3 vs. Q1, OR = 0.48, 95% CI: 0.31, 0.75), while moderate intake of C20:0 was directly associated with depressive symptoms (Q3 vs. Q1, OR = 1.87, 95% CI: 1.26, 2.77). Regarding single MUFAs, higher C14:1 intake was directly associated with depressive symptoms (Q4 vs. Q1, OR = 1.71, 95% CI: 1.12, 2.61), while moderate intake of C22:1 was associated with lower odds of having depressive symptoms (Q3 vs. Q1, OR = 0.54, 95% CI: 0.33, 0.86).

Finally, among single PUFAs, individuals with moderate and higher intakes of C20:4 were less likely to have depressive symptoms (OR = 0.64, 95% CI: 0.45, 0.91; OR = 0.59, 95% CI: 0.38, 0.91, respectively). Moreover, higher C20:5 intake was inversely associated with depressive symptoms (Q4 vs. Q1, OR = 0.35, 95% CI: 0.12, 0.98), as was low intake of C22:6 (Q2 vs. Q1, OR = 0.33, 95% CI: 0.12, 0.88).

## 4. Discussion

The current study aimed to test the relationship between dietary fat intake and depressive symptoms in Italian subjects living in a Mediterranean region. Although SFAs have always been considered detrimental to mental health, in our cohort, only moderate SFA consumption was associated with depressive symptoms. In fact, previous studies on animals showed that high intake of these fatty acids stimulates pro-inflammatory cytokine production and apoptosis in astrocytes, thus determining neuroinflammation that, in turn, impairs the dopamine system and increases the risk of depression [31]. Moreover, a diet high in SFAs lowered brain-derived neurotrophic factor (BDNF) levels in rodents, which is a factor in neurotransmission, neuronal survival growth, and plasticity [39]. Evidence from a longitudinal Australian older-adult cohort showed a positive association between SFA intakes and depressive symptoms mediated by C-reactive protein, suggesting a critical role of inflammation on the onset of depression [40]. In fact, inflammatory markers may affect the metabolism of main neurotransmitters involved in mood regulation, while an increase in secretion of pro-inflammatory cytokines may have an influence on the hypothalamic-pituitary-adrenal axis and thus on cortisol production, which contributes to depressive symptoms [41]. A recent prospective study showed that baseline SFA intake predicted depressive symptoms in women at midlife [26], while another cross-sectional study failed to find a statistically significant association after adjustment for total calories [27]. Only a randomized crossover study on healthy subjects tested the effects of consuming a diet high in SFAs for 4 days, showing that depression scores did not change significantly compared to a low-fat or PUFA-rich diet [42]. A probable reason for these debatable results may be attributable to the type of SFA mainly consumed in the diet. In fact, the different lengths of the carbon chains of each saturated fatty acid determine its absorption and metabolic effect [43]. For example, LCSFAs such as palmitate are known to increase cell membrane fluidity and flexibility, thus altering receptor functioning and leading to changes in lipid raft composition, which in turn contribute to polarization of the microglia and increase production of pro-inflammatory cytokines [44]. Interestingly, in our cohort, we found a positive association between an LCSFA of C20:0 and depressive symptoms, while individuals consuming low and moderate amounts of SCSFA were less likely to have depressive symptoms. SCSFAs (i.e., butyric acid and propionic acid) are mainly derived from the fermentation of dietary fiber in the colon, but they are also originally found in milk and whole dairy products [44]. The relevant role of SCSFAs in depression is suggested by studies that have found decreased levels of SCSFAs in the fecal samples of depressed women [45] and that their exogenous administration showed to reduce depressive symptoms in mice [46]. There are many mechanisms by which SCSFAs may influence depression; first of all, they ameliorate intestinal barrier integrity by strengthening tight junctions [47]. It is hypothesized that mood disorders are associated with dysbiosis with the consequent secretion of lipopolysaccharide (LPS) endotoxin into plasma, which, when combined with altered gut barrier integrity, induces systemic inflammatory effects in the brain [48]. In this context, SCSFA may exert anti-inflammatory effects by inhibiting NF-kB, the principal pro-inflammatory cytokine [49]. Moreover, they act on neuroinflammation by modulating microglia activation [50] and enhancing N-methyl-D-aspartate receptor activity [51]. SCSFAs may also influence the risk of depression by inhibiting the function of histone deacetylase, thus having an epigenetic effect [52]. Finally, SCSFAs bind and activate G protein-coupled receptors and influence the secretion of neurotransmitters such as 5-HT and acetylcholine by enterochromaffin cells [53]. To date, this is the first study examining the effects of each fatty acid subcategory on depression, although a recent systematic review of observational studies regarding the effects of one of the main dietary food sources of SFA, dairy products, on depression showed conflicting and inconsistent results [54]. This finding indicates that, although dairy products are rich in SFAs, their content of SCSFAs, particularly butyric acid, fat-soluble vitamins, and beneficial phospholipids can mitigate their potential adverse health effects [55].

In regard to MUFAs, few studies have investigated their role in depression. The SUN prospective cohort showed an inverse, weakly significant dose-response relationship for MUFAs on depression [9], while data on the protective effects of oleic acid (C18:1) are more consistent [56]. Oleic acid represents the most common MUFA that is largely contained in extra virgin olive oil, a cornerstone of the Mediterranean diet. Data obtained from healthy subjects who had a diet rich in MUFA from olive oil for years showed amelioration in hippocampal functions, and this improvement was linked with a lower incidence of depression [57]. MUFAs may ameliorate brain membrane fluidity through the increase of delta-9 desaturase enzyme activity, which in turn facilitates the binding of serotonin to its receptors [58]. Moreover, olive oil also contains other bioactive compounds, such as tyrosol, with relevant anti-inflammatory and antidepressant properties [59], which may explain the pleiotropic effect of this food rather than the individual intake of oleic acid. In our cohort, we found a null effect for oleic acid while high levels of myristoleic acid (C14:1) were directly associated with depressive symptoms, but little is known about this fatty acid effect. Moreover, subjects with moderate intakes of erucic acid (C22:1) were less likely to have depression. Regarding erucic acid, past studies on animals were alarming because it appeared to have toxic effects on the cardiovascular system. However, these detrimental effects have never been observed in humans, and indeed, some Asian populations usually consume this fatty acid without any reported toxicity [60]. Interestingly, a recent review reported that erucic acid may ameliorate cognition by interacting with peroxisome proliferator-activated receptors (PPARs) and inhibiting thrombin and elastase [61].

Among all these fatty acid categories, PUFAs are the ones that have been most studied in mood and cognitive disorders, starting from the consideration that they are notably abundant in the membranes of the brain and are also critical for neurodevelopment [62]. In line with the previous literature, our results showed that individuals with a higher consumption of n-3 PUFAs, especially eicosapentaenoic acid (EPA, C20:5), were less likely to have depression. Epidemiological studies have found that low circulating levels of n-3 PUFAs have been linked to major depressive disorder and prenatal depression associated with preterm birth [63,64]. Moreover, a recent umbrella review confirmed the role of n-3 PUFAs, especially from fish, in the prevention of depression [65]. Recognizing the anti-inflammatory effects and their role in the maintenance of membrane integrity and fluidity, EPA and DHA were also considered as adjuvant therapies for the treatment of depression [66,67]. There are multiple pathways by which PUFA may have an effect on the central nervous system and thus reduce the risk of depression. Firstly, they act directly on serotonin, beta-adrenergic, and dopamine receptor signaling, influencing mood [68]. Secondarily, they play a crucial role in reducing neuroinflammation, which is a chronic inflammatory state that is strongly associated with neurodegenerative diseases [69]. In fact, n-3 PUFAs, especially EPA, inhibit microglia activation and consequently the secretion of proinflammatory cytokines [70], while increasing TGF-β1 production [71]. Moreover, DHA may act as an immunomodulator, blocking the LPS-derived neuroinflammation and restoring synaptic functions in hippocampal CA1 pyramidal neurons [72]. Finally, both EPA and DHA may have anti-inflammatory properties through protectin and resolvin production [73], as well as through their competition for the enzymes regulating the synthesis of pro-inflammatory mediators from n-3 PUFAs [74]. In fact, previous evidence has sustained the assumption that n-3 and n-6 PUFAs have antagonistic roles on health, with the former playing an anti-inflammatory action, while the latter a pro-inflammatory effect due to the synthesis of negative eicosanoids from arachidonic acid metabolism and the reduction in the conversion of ALA into EPA or DHA [75]. Two recent meta-analyses showed that individuals with depressive symptoms had higher circulating levels of arachidonic acid than control subjects [76] and that a high ratio of n-6/n-3 PUFAs in their diet was linked to depressive symptoms [77]. Surprisingly, in our cohort, moderate and higher intakes of arachidonic acid (ARA, C20:4) were inversely associated with depressive symptoms. Although much of the previous scientific literature suggests that higher intake of dietary n-6 PUFAs, especially ARA, may exacerbate and dysregulate the inflammatory response in the nervous system [78], contradictory data have been reported when considering human studies [23], which are further supported by data from clinical trials providing null effects [79]. An n-6 PUFA deficiency was associated with lower cerebral content of dopamine and serotonin [80], while a higher intake was associated with an increase in BDNF [81]. In a cross-sectional study, Japanese male adults with higher ARA intake were less likely to have depression [82]. In fact, moderate ingestion of ARA may have a potentially beneficial role on neurons activating brain cannabinoid receptors, protecting from oxidative stress and ameliorating membrane fluidity and synaptic plasticity in the hippocampus [83]. Further investigation on the effects of dietary n-6 PUFAs on the nervous system is still needed to better understand neurodegenerative disease and depression.

The result of the present investigation may be subjected to some limitations. First, the cross-sectional design does not allow for the definition of causality between variables; thus, the results may be affected by reverse causation. Furthermore, although we carried out multivariate-adjusted logistic regression analyses, residual confounding cannot be ruled out. Another limitation regards the nutritional assessment method: FFQs may underestimate or overestimate food intake due to recall bias, portion size miscalculation, and social desirability bias. Finally, the CSE-D is able to assess depressive symptoms but should not be considered equal to a clinical diagnosis of depression. Further studies with a prospective approach are needed to thoroughly investigate the role of dietary fat on depression.

## 5. Conclusions

Although over the years dietary fatty acids have been blamed to be the main nutritional risk factor for the increase in non-communicable disease, the findings of our study underline the importance of differentiating each category of fats according to the length of their carbon chain. In our study, we found a positive association between LCSFA and depressive symptoms, while subjects consuming SCSFA were less likely to have depressive symptoms. Among PUFAs, in line with previous literature, the intake of EPA was negatively associated with depressive symptoms. In addition, moderate ingestion of ARA seems to be beneficial for mental health, as opposed to the belief that it may be pro-inflammatory. In conclusion, this study confirms the pivotal role of PUFAs and reopens the debate on the role of saturated fatty acids, suggesting plausible beneficial effects of moderate intakes of short-chain fatty acids, which act on inflammation and the gut-brain axis. Further studies with a prospective approach and a clinical diagnosis of depression are needed to thoroughly investigate the role of each dietary fat category on depression.

## Figures and Tables

**Table 1:** Background characteristics of the study sample by consumption of total dietary fats (*n* = 1572).

	Total Fats				*p*-Value
	Q1 (*n* = 419)	Q2 (*n* = 424)	Q3 (*n* = 360)	Q4 (*n* = 369)	
**Sex, *n* (%)**					0.062
Male	169 (40.3)	200 (47.2)	150 (41.7)	141 (38.2)	
Female	250 (59.7)	224 (52.8)	210 (58.3)	228 (61.8)	
**Age, mean (SD)**	48.9 (18.1)	48.65 (17.2)	44.6 (16.5)	43.7 (16.3)	<0.001
**Educational status, *n* (%)**					<0.001
Low	133 (31.7)	130 (30.7)	75 (20.8)	119 (32.2)	
Medium	138 (32.9)	176 (41.5)	176 (48.9)	154 (41.7)	
High	148 (35.3)	118 (27.8)	109 (30.3)	96 (26.0)	
**Smoking status, *n* (%)**					0.189
Non-smoker	288 (68.7)	270 (63.7)	225 (62.5)	227 (61.5)	
Current smoker	93 (22.2)	110 (25.9)	92 (25.6)	89 (24.1)	
Former smoker	38 (9.1)	44 (10.4)	43 (11.9)	53 (14.4)	
**Physical activity level, *n* (%)**					0.001
Low	81 (19.4)	77 (18.2)	43 (12.0)	76 (20.6)	
Medium	202 (48.4)	183 (43.3)	204 (56.8)	186 (50.1)	
High	134 (32.1)	163 (38.5)	112 (31.2)	108 (29.3)	
**BMI categories, *n* (%)**					0.311
Normal	208 (50.5)	187 (45.7)	158 (48.3)	147 (48.2)	
Overweight	129 (31.3)	148 (36.2)	122 (37.3)	115 (37.7)	
Obese	75 (18.2)	74 (18.1)	47 (14.4)	43 (14.1)	
**Mediterranean diet adherence, *n* (%)**					<0.001
Low	267 (63.7)	223 (52.6)	197 (54.7)	171 (46.3)	
Medium	128 (30.5)	145 (34.2)	136 (37.8)	148 (40.1)	
High	24 (5.7)	56 (13.2)	27 (7.5)	50 (13.6)	

**Table 2:** Association between total and dietary fat classes and depressive symptoms in the study sample (*n* = 1572).

	OR (95% CI)			
	**Q1**	**Q2**	**Q3**	**Q4**
**Total fats**				
Energy-adjusted	1	0.86 (0.64, 1.17)	1.18 (0.85, 1.63)	1.32 (0.87, 2.01)
Multivariate-adjusted	1	0.93 (0.68, 1.28)	1.23 (0.87, 1.75)	1.28 (0.80, 2.03)
**Saturated fats**				
Energy-adjusted	1	0.99 (0.73, 1.33)	1.41 (1.02, 1.95)	1.16 (0.77, 1.75)
Multivariate-adjusted	1	1.01 (0.74, 1.39)	1.44 (1.02, 2.04)	0.95 (0.59, 1.53)
**MUFA**				
Energy-adjusted	1	0.74 (0.54, 0.99)	1.16 (0.83, 1.61)	1.56 (1.03, 2.36)
Multivariate-adjusted	1	0.76 (0.55, 1.04)	1.22 (0.86, 1.73)	1.52 (0.97, 2.38)
**PUFA**				
Energy-adjusted	1	0.59 (0.44, 0.80)	0.77 (0.55, 1.07)	0.85 (0.54, 1.32)
Multivariate-adjusted	1	0.60 (0.44, 0.84)	0.81 (0.57, 1.16)	0.88 (0.54, 1.44)

Multivariate models were adjusted for age, sex, BMI, educational level, smoking status, physical activity level, total energy intake, and adherence to the Mediterranean diet.

**Table 3:** Association between specific fats and depressive symptoms in the study sample (*n* = 1572).

	OR (95% CI)			
	**Q1**	**Q2**	**Q3**	**Q4**
**Saturated fats**				
C4-C10	1	0.62 (0.43, 0.89)	0.48 (0.31, 0.75)	0.72 (0.45, 1.14)
C12:0	1	1.08 (0.73, 1.61)	0.84 (0.52, 1.37)	1.22 (0.70, 2.11)
C14:0	1	1.23 (0.80, 1.87)	1.72 (0.95, 3.11)	1.84 (0.85, 4.00)
C16:0	1	1.35 (0.77, 2.36)	1.14 (0.52, 2.53)	0.77 (0.27, 2.19)
C18:0	1	0.66 (0.37, 1.68)	0.90 (0.37, 1.17)	0.78 (0.30, 2.00)
C20:0	1	0.87 (0.62, 1.22)	1.87 (1.26, 2.77)	1.65 (0.96, 2.82)
C22:0	1	1.00 (0.72, 1.40)	0.86 (0.60, 1.25)	0.86 (0.55, 1.37)
**MUFA**				
C14:1	1	1.24 (0.90, 1.70)	1.26 (0.87, 1.82)	1.71 (1.12, 2.61)
C16:1	1	0.71 (0.49, 1.03)	0.78 (0.48, 1.28)	0.61 (0.32, 1.14)
C18:1	1	0.85 (0.59, 1.23)	1.40 (0.87, 2.24)	1.58 (0.87, 2.84)
C20:1	1	0.97 (0.69, 1.37)	0.78 (0.48, 1.25)	1.84 (0.92, 3.72)
C22:1	1	1.39 (0.99, 1.94)	0.54 (0.33, 0.86)	0.53 (0.27, 1.58)
**PUFA**				
C18:2	1	0.81 (0.55, 1.21)	1.06 (0.66, 1.69)	0.89 (0.49, 1.62)
C18:3	1	0.96 (0.65, 1.42)	1.25 (0.78, 2.02)	1.27 (0.72, 2.25)
C20:4	1	0.79 (0.58, 1.07)	0.64 (0.45, 0.91)	0.59 (0.38, 0.91)
C20:5	1	0.56 (0.35, 0.91)	0.26 (0.12, 0.56)	0.35 (0.12, 0.98)
C22:6	1	0.33 (0.12, 0.88)	0.58 (0.24, 1.43)	0.77 (0.38, 1.53)

All analyses were adjusted for age, sex, BMI, educational level, smoking status, physical activity level, total energy intake, and adherence to the Mediterranean diet.

## Data Availability

The data that support the findings of this study are available upon reasonable request.
